# Biomechanical Study of a Novel, Expandable, Non-Metallic and Radiolucent CF/PEEK Vertebral Body Replacement (VBR)

**DOI:** 10.3390/ma12172732

**Published:** 2019-08-26

**Authors:** Daniel Adler, Michael Akbar, Anna Spicher, Stephanie-Alice Goerke, Werner Schmoelz

**Affiliations:** 1 Spine Center, Department of Orthopaedic Surgery, Trauma Surgery and Division of Spinal Cord Injury, Ruprecht-Karls-University Heidelberg, Schlierbacher Landstraße 200A, 69118 Heidelberg, Germany; 2 Department of Trauma Surgery, Medical University of Innsbruck, Innrain 52, 6020 Innsbruck, Austria; 3 Department of Anatomy, Medical University of Innsbruck, Innrain 52, 6020 Innsbruck, Austria

**Keywords:** Vertebral body replacement (VBR), non metallic, radiolucent, CF/PEEK, biomechanics, tumor, vertebral fracture, spine

## Abstract

Vertebral body replacement is well-established to stabilize vertebral injuries due to trauma or cancer. Spinal implants are mainly manufactured by metallic alloys; which leads to artifacts in radiological diagnostics; as well as in radiotherapy. The purpose of this study was to evaluate the biomechanical data of a novel carbon fiber reinforced polyetheretherketone (CF/PEEK) vertebral body replacement (VBR). Six thoracolumbar specimens were tested in a six degrees of freedom spine tester. In all tested specimens CF/PEEK pedicle screws were used. Two different rods (CF/PEEK versus titanium) with/without cross connectors and two different VBRs (CF/PEEK prototype versus titanium) were tested. In lateral bending and flexion/extension; range of motion (ROM) was significantly reduced in all instrumented states. In axial rotation; the CF/PEEK combination (rods and VBR) resulted in the highest ROM; whereas titanium rods with titanium VBR resulted in the lowest ROM. Two cross connectors reduced ROM in axial rotation for all instrumentations independently of VBR or rod material. All instrumented states in all planes of motion showed a significantly reduced ROM. No significant differences were detected between the VBR materials in all planes of motion. Less rigid CF/PEEK rods in combination with the CF/PEEK VBR without cross connectors showed the smallest reduction in ROM. Independently of VBR and rod material; two cross connectors significantly reduced ROM in axial rotation. Compared to titanium rods; the use of CF/PEEK rods results in higher ROM. The stiffness of rod material has more influence on the ROM than the stiffness of VBR material.

## 1. Introduction

Vertebral body replacement (VBR) combined with dorsal instrumentation is the preferred treatment option to achieve decompression and restore stability of the spinal column either in patients with a traumatic fracture (>AO Spine A3) [1,2,3,4,5,6] or major pathological instability due to infection [7,8,9,10], spinal metastases [11,12,13] or primary malignancy [7,8,11,12,13]. Metastatic spinal lesions occur in 5%–10% of all cancer patients while primary spinal tumors are a rare entity [12]. Most spinal metastases are located anteriorly in the vertebral body and in the peridural space, while a dorsal location is rare [4,6,10,11,12,14]. The incidence of clinically apparent metastatic disease in the spine has increased since advances in tumor treatment generally have improved the life expectancy of cancer patients [10,15]. Local tumor progression can lead to vertebral body collapse with decompensation of the sagittal profile, causing pain and neurological deficits [10,15,16]. Surgical treatment options are mainly palliative and adapted to the patients’ general condition, aiming for minimal peri- and postoperative morbidity [10,15]. However, more radical treatment options with removal of spinal lesions showed better results in patients with good prognosis [17,18,19,20]. A loss of correction [4,7,8] or implant failures due to loosening at the implant–bone interface with recurrent instability [1,9,10,13,16] are well described after isolated posterior instrumentation. 360° instrumentations provide the highest stiffness in biomechanical studies, where a rotationally unstable corpectomy defect model is used [4,6,7,10]. Postoperative local radiotherapy and chemotherapies may complicate the postoperative course by prolonged instability with wound infections (threefold higher risk), hardware failure, or progressive implant loosening [21,22]. In tumor patients, general condition and life expectancy have to be taken into account. Patients with prolonged life expectancy (>12 months) are recommended to be treated with a 360° instrumentation [15,17,18,19,20]. To provide sufficient rotational stability and load-bearing capacity, the vast majority of common VBRs are manufactured from different metallic alloys, preferably titanium. But imaging artifacts due to the presence of metal components in CT or MRI imaging adversely complicates postoperative radiological diagnostics in local tumor recurrence or local radiation therapy. High strength, non-metallic dorsal and ventral implants made from carbon fiber reinforced polyetheretherketone (CF/PEEK) have recently become available to avoid these artifacts [10]. Precise knowledge of biomechanical characteristics (VBR combined with dorsal instrumentation) is essential for safe clinical application and long-term spinal stability [10,11]. 

Various biomechanical [1,4,5,6,11,12] and clinical studies [2,3,23,24] have addressed spinal stability after implantation of a metallic VBR, while biomechanical studies evaluating non-metallic VBR are rare. The objective of the present study was to quantitatively analyze the biomechanical data of a new, non-metallic CF/PEEK VBR in combination with a dorsal CF/PEEK screw and rod system, with and without cross connectors. The implant combinations with the novel material were compared to a common titanium VBR in combination with titanium rods. Construct stiffness and flexibility (range of motion (ROM)) were tested in a six degrees of freedom spine simulator. To our best knowledge, this is the first study to evaluate the biomechanical characteristics of an in-situ, expandable, non-metallic, radiolucent VBR in combination with non-metallic, radiolucent posterior pedicle screw instrumentation.

## 2. Materials and Methods

Six (*n* = 6) fresh frozen human thoracolumbar spine units (Th11–L3) were tested with a mean age at death of 59.2 years (ranging from 56 to 65 years). The bodies were donated by people who had given their written informed consent prior to death to use their bodies for scientific and educational purposes. For standardization and homogeneous study conditions, all tested vertebral bodies were analyzed via pre-interventional CT scan (General Electrics, Lightspeed VCT 16, qCT including EFP calibration, GE Medical Systems, USA). Trabecular bone mineral density (BMD) was measured between 66.8 mg/ccm and 100.4 mg/ccm (mean BMD: 82.0 mg/ccm, SD: ± 27.6). Spinal specimens with deformities, previous spinal surgery, structural disorders or post traumatic disorders were excluded. The vacuum sealed, frozen specimens (minus 20 °C) were thawed overnight at 6 °C before all soft tissues were removed, preserving supporting spinal ligaments and joint capsules. 

**Figure 1:**
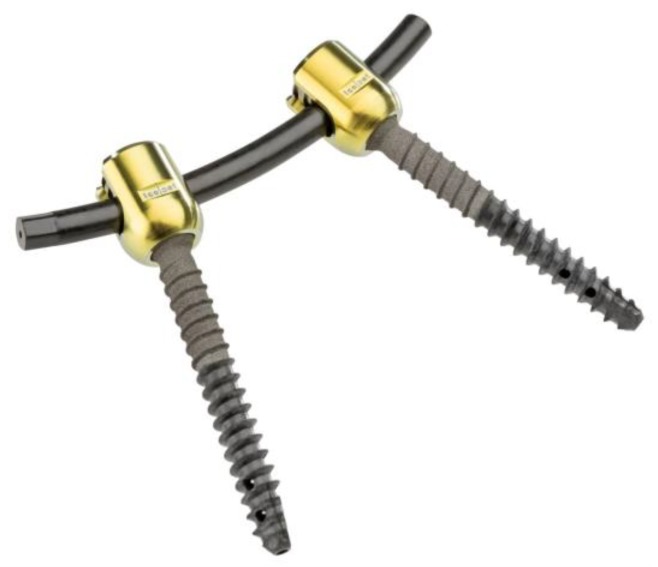
Non-metallic X-ray-translucent carbon fiber reinforced polyetheretherketone (CF/PEEK) pedicle screws (icotec: VADER^®^, Altstätten, Switzerland).

For posterior instrumentation, pedicle entry points were identified according to the anatomical landmarks and controlled using bi-planar fluoroscopy. Nonmetallic, radiolucent 6.5 × 45 mm CF/PEEK pedicle screws (VADER^®^, icotec, Altstätten, Switzerland) were inserted in all specimens at the Th11/12 level, as well as at the L2/3 level for all investigated cases (Figure 1). Pedicle screws were combined with two different rod types in the various cases tested: A nonmetallic and radiolucent 6 mm CF/PEEK rod (icotec: VADER^®^, Altstatten, Switzerland) and a 6.0 mm standard titanium rod system (icotec ag, Altstätten, Switzerland). Titanium cross connectors (icotec ag, Altstätten, Switzerland) completed the setup.

**Figure 2:**
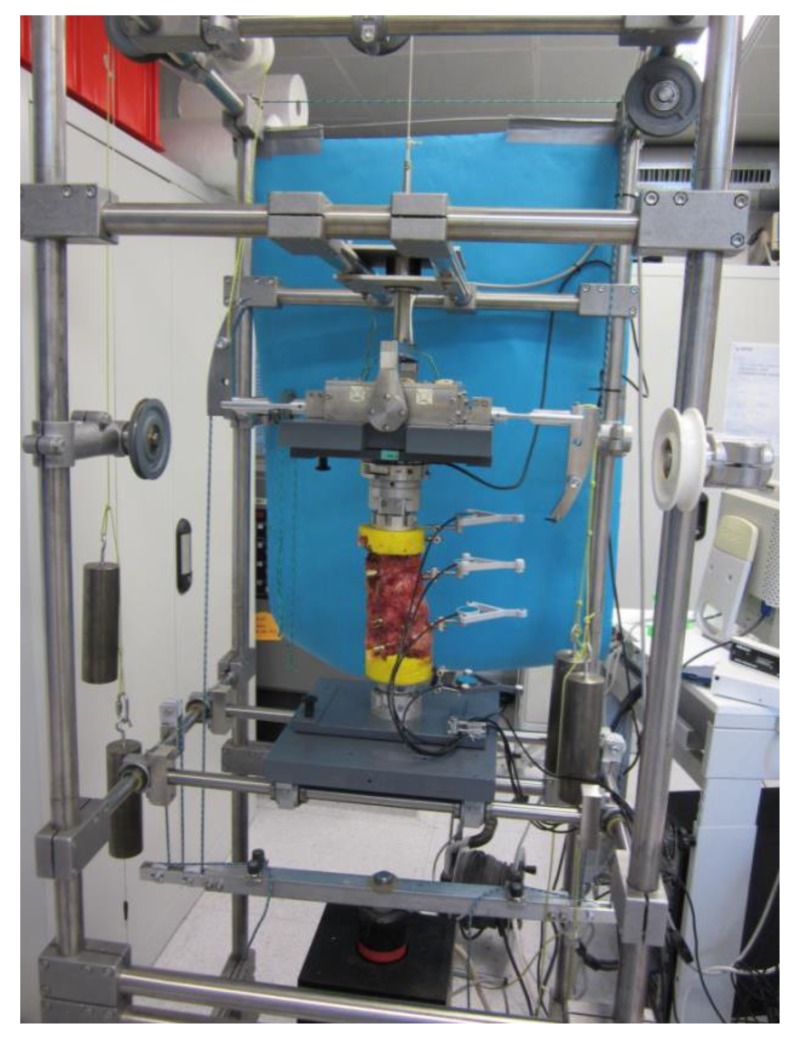
Spine tester (Schunk FT Delta SI 660-60, Lauffen/Neckar, Germany) with six degrees of freedom; three in translation (green) and three in rotation (orange). Specimen embedded in PMMA.

The cranial (TH11) and the caudal vertebra (L3) were embedded in polymethylmethacrylate (PMMA: Heraeus Kulzer GmbH, Technovit 3040, Wehrheim, Germany) with sufficient clearance for pedicle instrumentation and with the midline of all five vertebrae aligned horizontally. Flanges in the upper and lower PMMA blocks provided a rigid fixation to the spine simulator. To measure intersegmental motion of the treated (Th12–L2) and adjacent segments (Th11/12 and L2/3), an ultrasound-based 3D motion analysis system (Winbiomechanics, Zebris, Isny, Germany, resolution 0.1°) was fixed to the ventral side of the vertebrae (Figure 2). According to international standards, all tests were carried out at room temperature and specimens were kept moist with isotonic saline solution for the study period [25,26,27,28]. With respect to the recommendations for testing of spinal implants [25,26,27,28], biomechanical testing was performed in a six degrees of freedom spine simulator as described by Knop et al. [6] and Schmoelz et al. [29], equipped with a six-component load cell (Schunk FT Delta SI 660-60, Lauffen/Neckar, Germany) with feedback control and a connection to a stepper motor for load application (Figure 2). For all tests, pure moments of ±7.5 Nm were applied in the three main motion planes: Flexion/extension, lateral bending (left/right) and axial rotation (left/right).

**Figure 3:**
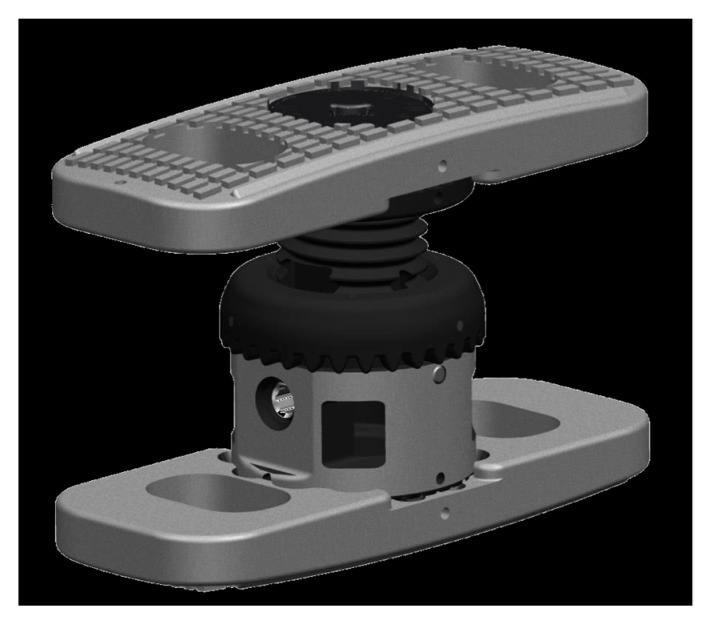
Prototype of the non-metallic, X-ray-translucent CF/PEEK expandable VBR. icotec, Altstätten, Switzerland.

At first, the intact specimens (Th11-L3) were loaded with pure moments of 7.5 Nm to record a baseline. Afterwards a corpectomy of the target vertebra L1 with removal of the cranial and caudal discs was conducted with standard surgical tools, according to clinical routine. For the reconstruction of the anterior spinal column, either an expandable, nonmetallic radiolucent CF/PEEK VBR (prototype, icotec Altstätten, Switzerland) (Figure 3) or an expandable titanium VBR (X-Core, Nuvasive, Bremen, Germany) was implanted. Both VBRs consist of an in situ expandable centerpiece, to which modular endplates can be attached. While the CF/PEEK VBR prototype is nonmetallic and radiolucent with macrostructured endplates to prevent dislocation, the X-Core VBR is manufactured from titanium alloy and its endplates are equipped with spikes to prevent dislocation. For both implants, height can be adjusted continuously within a clinically relevant range by a gear wheel drive unit. The desired height can be locked in position using a locking screw. The VBR endplate´s size was determined via templates and the VBR was placed in typical technique.

**Figure 4:**
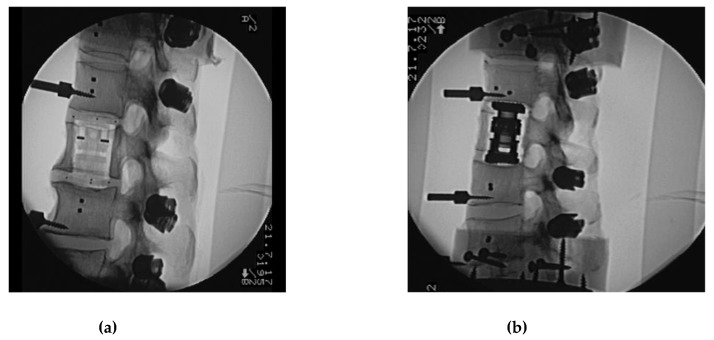
Lateral view native radiographs documenting correct positioning of the (**a**) prototype non-metallic, X-ray-translucent CF/PEEK expandable vertebral body replacement (VBR) and the expandable titanium VBR (**b**).

All surgical procedures were performed by the first author (experienced senior spine surgeon). Two plane (anterior, posterior and lateral view) native radiographs were taken to control and document correct positioning of pedicle screws and VBRs (Figure 4a,b). Final fixation of posterior rods to the pedicle screws was carried out in a standardized fashion with an axial preload of the spine simulator of 100 N.

## 3. Study Protocol

For each specimen, the following states were tested with alternatives for the VBR (CF/PEEK or titanium), the posterior rod material (CF/PEEK or titanium), and the application of cross connectors.

(1) Flexibility test, Native (**native**):
*Corpectomy and instrumentation with VBR and pedicle fixation with CF/PEEK rod.*
(2) Flexibility test—CF/PEEK *VBR* instrumented with *CF/PEEK rod* (**CF_CF**):
*Change of posterior rod fixation to titanium rod.*
(3) Flexibility test—CF/PEEK *VBR* Instrumented with *titanium rod*, (**CF_Ti**):
*Change of VBR to titanium.*
(4) Flexibility test—titanium *VBR* instrumented with *titanium rod* (**Ti_Ti**):
*Addition of two cross connectors to the posterior rods dissecting the ligamentum supraspinale/interspinale.*
(5) Flexibility test—titanium *VBR* instrumented with *titanium rod* and 2 cross connectors (**Ti_Ti_cc**):
*Change to CF/PEEK VBR.*
(6) Flexibility test—*CF/PEEK VBR* instrumented with *titanium rod* and 2 cross connectors (**CF_Ti_cc**):
*Change rods to CF/PEEK.*
(7) Flexibility test—*CF/PEEK VBR* Instrumented with *CF/PEEK rod* and 2 cross connectors (**CF_CF_cc**).

Statistical analysis of the ROM was performed using the SPSS software (Microsoft Windows release 24, SPSS Inc., Chicago, IL, USA). Data and results of the ROM were evaluated for the three motion directions, normalized and compared to the motion of the native segment. A general linear model (GLM) with repeated measures was used for statistical comparison. *p*-values were calculated with adjustment for multiple corrections.

## 4. Results

**Figure 5:**
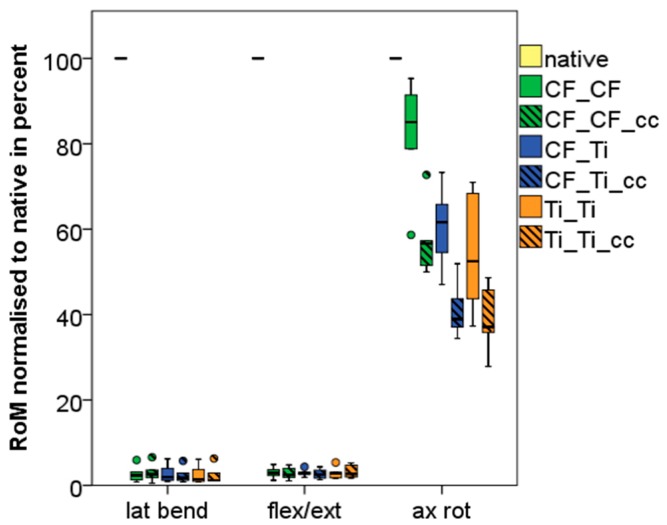
Boxplot showing the median and quartiles of the index segment for all tested states in the three motion directions normalized in percentage of the native state.

Results of the ROM are displayed in absolute metrics (Table 1) and as boxplot normalized to non-instrumented native condition (Figure 5) in the three planes of motion for the index segments (Th12–L2).

### 4.1. Index Segments (Th12-L2)

All instrumented states in all planes of motion showed a significantly (*p* < 0.05) reduced ROM compared to the native state. 

In lateral bending, the mean ROM in the native state was 11.2° (SD 3.26). The various instrumentations reduced ROM to 0.24°–0.30° (SD 0.18–0.21). No significant differences (<0.1°, *p* = 1.0) in ROM were measured for the different VBR material (CF/PEEK versus titanium), posterior rod material (CF/PEEK versus titanium) or additional cross connectors. 

In flexion/extension, the mean ROM in the native state was 10.3° (SD 2.4). Varying instrumentations reduced ROM to 0.29°–0.35° (SD 0.12–0.31). No significant differences (<0.1°, *p* = 1.0) in ROM were measured for the different VBR material (CF/PEEK versus titanium), posterior rod material (CF/PEEK versus titanium), or additional cross connectors.

In axial rotation the mean ROM in native state was 4.83° (SD 0.95). The various instrumentations reduced ROM to 1.84°–3.95° (SD 0.33–0.9). Less rigid CF/PEEK rods combined with CF/PEEK VBR without the use of cross connectors showed the smallest ROM reduction to 3.95° (SD 0.9) in axial rotation. Titanium rods with titanium VBR and the use of two cross connectors reduced ROM to 1.84° (SD 0.33). Independent of type of VBR or rod material (CF/PEEK or titanium), two additional cross connectors reduced significantly, (*p* < 0.05) the ROM in axial rotation. Posterior rod material (CF/PEEK or titanium) had greater effects to the ROM than VBR material (CF/PEEK or titanium) in axial rotation.

### 4.2. Caudal Adjacent Segment (L2/3)

Equally to the index level, all instrumented states showed in all planes of motion a significantly (*p* < 0.05) reduced ROM compared to native state. 

In lateral bending the mean ROM in native state was 7.9° (SD 2.64). The various instrumentations reduced ROM to 1.38°–1.76° (SD 0.66–1.02). No significant differences (<0.4°, *p* > 0.78) in ROM were measured for the different VBR materials (CF/PEEK versus titanium), posterior rod materials (CF/PEEK versus titanium) or additional cross connectors. Similar to the index segment, mean ROM of the L2/3 segment in lateral bending was slightly higher (0.2°) with the use of two cross connectors.

In flexion/extension the mean ROM in native state was 7.5° (SD 2.76). The various instrumentations reduced ROM to 1.13°–1.50° (SD 0.58–0.95). No significant differences in ROM were measured for the different VBR material (CF/PEEK versus titanium), posterior rod material (CF/PEEK versus titanium) or additional cross connectors. Similar to lateral bending mean ROM was slightly (0.2°) higher with the use of two cross connectors.

In axial rotation the mean ROM in native state was 3.88° (SD 2.33). The various instrumentations reduced ROM to 1.27°–1.96° (SD 0.48–0.85). Less rigid CF/PEEK rods combined with CF/PEEK VBR without the use of cross connectors again showed smallest ROM reduction to 1.96° (SD 0.85) in axial rotation. Titanium rods with titanium VBR and the use of two cross connectors reduced ROM to 1.27° (SD 0.48). Independently to VBR or rod material (CF/PEEK or titanium) two cross connectors significantly reduced ROM in axial rotation (*p* < 0.05). Posterior rod material (CF/PEEK or titanium) had greater effects to the ROM than VBR material (CF/PEEK or titanium) in axial rotation.

### 4.3. Cranial Adjacent Segment (Th11/12)

Equally to the index and caudal adjacent level, all instrumented states showed, in all planes of motion, a significantly (*p* < 0.05) reduced ROM compared to native state. 

In lateral bending the mean ROM in native state was 4.49° (SD 3.40). The various instrumentations reduced ROM to 2.61°–2.96° (SD 1.41–1.63). No significant differences in ROM were measured for the different VBR material (CF/PEEK versus titanium), posterior rod material (CF/PEEK versus titanium) or additional cross connectors. Similar to the index and caudal adjacent level mean ROM of Th11/12 segment was slightly (0.3°) higher with the use of two cross connectors in lateral bending.

In flexion/extension the mean ROM in native state was 6.59° (SD 2.80). The various instrumentations reduced ROM to 1.87°–2.28° (SD 1.34–1.90). No significant differences in ROM were measured for the different VBR material (CF/PEEK versus titanium), posterior rod material (CF/PEEK versus titanium) or additional cross connectors. Similar to lateral bending the mean ROM was slightly (0.3°) higher with the use of two cross connectors in lateral bending.

In axial rotation the mean ROM in native state was 4.27° (SD 1.93). The various instrumentations reduced ROM to 2.70°–3.25° (SD 1.27–1.47). Less rigid CF/PEEK rods combined with CF/PEEK VBR without the use of cross connectors again showed smallest ROM reduction to 3.25° (SD 1.45) in axial rotation. Titanium rods with titanium VBR and the use of two cross connectors reduced ROM to 2.70° (SD 1.42). Independently to VBR or rod material (CF/PEEK or titanium) two cross connectors reduced (*p* < 0.05) ROM in axial rotation slightly. Posterior rod material (CF/PEEK or titanium) had greater effects on the ROM than VBR material (CF/PEEK or titanium) in axial rotation.

## 5. Discussion

This is the first report of a biomechanical testing series utilizing a novel, nonmetallic, radiolucent and expandable CF/PEEK VBR for 360° instrumentation in combination with an established screw and rod system (rod material CF/PEEK and titanium). For this purpose a human cadaveric corpectomy defect model was tested in a spine tester with three-dimensional motion measurement of each segment. The implants presented in this study were designed to reconstruct spinal stability after corpectomy in traumatic or malignant vertebral fractures. Reconstruction was varied by anterior VBR material (CF/PEEK versus titanium) and dorsal instrumentation’s material (CF/PEEK versus titanium) with or without additional cross connectors. 

Our results indicate the less rigid CF/PEEK rods combined with CF/PEEK VBR without the use of cross connectors reduces the ROM in axial rotation by only 18% (γ = 3.95°, SD 0.9) compared to an intact specimen (γ = 4.83°, SD 0.95). In all other modes (flexion/extension and lateral bending) the CF/PEEK rods combined with CF/PEEK VBR provided comparable reduction in ROM when compared to titanium rods in combination with a titanium VBR.

Titanium rods in combination with a titanium VBR and the use of two cross connectors demonstrated significantly more stiffness in axial rotation with a decrease of ROM of 62% (γ = 1.84°, SD 0.33). Independently to VBR or rod material (CF/PEEK or titanium) two cross connectors significantly reduced (*p* < 0.05) the ROM in axial rotation. Posterior rod material (CF/PEEK or titanium) had greater effects on the ROM than VBR material (CF/PEEK or titanium) in axial rotation (Table 1, Figure 5). In lateral bending and flexion/extension varying instrumentations significantly (*p* < 0.05) reduced mean ROM by 97% and 96% compared to the intact specimen, respectively. There were no significant differences between different VBR and posterior rod system (CF/PEEK versus titanium).

Different studies [11,12,29,30,31] evaluated the biomechanical behavior of VBRs in combination with screw and rod systems. The length of the posterior instrumentation was shown to be the major determinant for the constructs’ stability/stiffness [30,31]. Longer posterior instrumentation (two adjacent levels, cranial and caudal to the VBR) provided significant higher stiffness compared to bi-segmental instrumentation even with an additional antero-lateral plate. Therefore, only posterior instrumentations with two adjacent levels above and below the VBR were tested in this study. Further studies [4,5,6,7,8,13] compared the stability of different in situ expandable VBRs and non-expandable VBRs in combination with/without posterior screw and rod systems and/or anterior instrumentation with a locked angular stable plate. Isolated anterior instrumentation (VBR and an antero-lateral plate) revealed a significantly lower stiffness compared to intact specimens. A significant increase of stiffness in all motion planes was detected after additional posterior screw and rod instrumentation [4,5,6,7,8,13]. Isolated anterior instrumentation (VBR combined with an additional anterior polyaxial or angular stable plating) cannot be recommended for stabilization of vertebral corpectomy defects [4,5,6,7,8,13]. In clinical routine, isolated anterior spinal instrumentation in metastatic disease to the spine is inappropriate. Consistent with other authors [4,6,7,8,12,23] we recommend either posterior instrumentation with/without decompression (patients with limited life expectancy or bad general condition) or a 360° instrumentation in patients with adequate general condition and life expectancy.

Carbon fiber reinforced polymer intervertebral implants were already described by Brantigan and Steffee in 1991 with excellent biomechanical results and fusion rates [32,33]. Schulte et al. [10] described a vertebral body replacement with a bioglass-polyurethane spacer fixed with a ventral plate of carbon-fiber reinforced polyethererherketone (CF-PEEK). Biomechanical testing detected a significant reduction of ROM in all three motion planes. In the course of the study, one patient died 18 months post operation. After biopsy and biomechanical testing of the explanted spinal segments the ROM in all motion planes demonstrated values comparable to the previous biomechanical testing with cadavers. Early signs of osteointegration at the bone-endplate interface in combination with mechanical interlocking by bony heterotypic ossifications resulted in an even improved stability. In comparison to titanium implants this osseous integration might provide additional stability in CF/PEEK implants.

Various non-expandable and expandable VBRs were tested [8,34,35] on primary stiffness. In 360° instrumentations the material of VBR was shown to have a minor effect in the treated segment. Anterior PMMA constructs [35], as well as titanium mesh cages [34], in combination with multilevel posterior instrumentation provided higher stiffness than intact specimens. Consistent to these results in the present study, no significant differences between the VBR materials (CF/PEEK versus titanium) were detected. 

360° instrumentations are proven to be biomechanically superior to isolated posterior instrumentations regarding stability and stiffness in treated spinal segments [8,31,34,36]. But it has to be kept in mind that an additional ventral stabilization increases surgical risk factors like an enlarged surgical approach, higher blood loss, increased risk of infection, and prolonged operation time [11]. Therefore, these procedures should be performed after carefully individualized decision making depending on the patient’s general condition, and only in experienced spine centers [11,16].

Common limitations of biomechanical in vitro testing also apply to the present study. Due to the lack of influence on biomechanical characteristics of vital spinal muscles, in vitro models are reduced to bony and ligamentary structures [11,34,35]. Tissue healing and consolidation of the bone as in vivo factors cannot be displayed and analyzed. Comparing the results with other studies is difficult, as variable testing conditions, sequences, and specimen characteristics (level, BMD, age, and species) can be found in the literature [11]. Another limitation is the relatively small sample size which was used to investigate the various reconstruction options. However, common inter-individual variables found in clinical routine can be excluded in the controlled laboratory environment. If the biomechanical effect of an intervention is not provable in controlled laboratory standards with a limited size of samples it is assumed rather unlikely to have clinical impact [37]. Conclusions concerning intermediate and long term stability of spinal reconstructions cannot be drawn from the present study as no cyclic loading was performed. Primary stability of the implants was determined with assessment of ROM using pure moments in a six degrees of freedom spine tester. The use of pure moments has well described limitations depending on the set-up features. Nevertheless, implant testing with pure moments is a worldwide [25,26,27,38] accepted method to compare various types of spinal instrumentations.

## 6. Conclusions

Compared to the native state, all instrumented states showed a significantly (*p* < 0.05) reduced ROM in all planes of motion. No significant differences were detected between the VBR materials (CF/PEEK versus titanium) in all planes of motion. Less rigid CF/PEEK rods in combination with the CF/PEEK VBR without cross connectors showed the smallest reduction in ROM. Independently of VBR and rod material (CF/PEEK versus titanium), two cross connectors significantly reduced ROM in axial rotation and are therefore highly recommended. Compared to titanium rods, the use of CF/PEEK rods results in higher ROM. The stiffness of rod material has more influence on the ROM than the stiffness of VBR material.

## Figures and Tables

**Table 1:** Results of the range of motion (ROM) test.

	Lateral Bending Th12-L2		Flexion/Extension Th12-L2		Axial Rotation Th12-L2	
	*Mean*	*SD*	*Mean*	*SD*	*Mean*	*SD*
Native	11.17	3.26	10.32	2,40	4.83	0.95
CF_CF	0.27	0.18	0.33	0.18	3.95	0.90
CF_CF_cc	0.30	0.21	0.30	0.20	2.74	0.46
CF_Ti	0.27	0.20	0.30	0.12	2.88	0.40
CF_Ti_cc	0.24	0.18	0.29	0.17	1.95	0.33
Ti_Ti	0.25	0.21	0.31	0.17	2.55	0.53
Ti_Ti_cc	0.24	0.21	0.35	0.22	1.84	0.33
